# Draft Genome Sequences of Multidrug-Resistant and *mcr-1.1*-Harboring Escherichia coli Isolated from Drinking and Well Waters Used in Syrian Refugee Camps

**DOI:** 10.1128/MRA.01252-20

**Published:** 2021-01-14

**Authors:** Nivin A. Nasser, Abdallah Alhaj Sulaiman, David Mann, Shaoting Li, Xiangyu Deng, Issmat I. Kassem

**Affiliations:** a Center for Food Safety, Department of Food Science and Technology, University of Georgia, Griffin, Georgia, USA; b Department of Nutrition and Food Sciences, Faculty of Agricultural and Food Sciences, American University of Beirut (AUB), Beirut, Lebanon; University of Maryland School of Medicine

## Abstract

Resistance to colistin, a last-resort antibiotic, threatens the treatment of complicated infections, especially in susceptible populations such as Syrian refugees who live in makeshift camps. Two multidrug-resistant Escherichia coli strains with the plasmid-borne colistin resistance gene (*mcr-1.1*) were isolated from the waters used by refugees and sequenced to analyze antibiotic resistance determinants.

## ANNOUNCEMENT

Many Syrian refugees live in makeshift camps with limited access to clean water, food, and proper sanitation (1–3). Previously, we showed that the refugees were exposed to multidrug-resistant Escherichia coli (4). These isolates were retrieved from composite water samples (1 liter) collected from the drinking reservoirs and wells that were located in the camps and used by the refugees. The water was filtered using 0.22-μm Millipore membranes that were then placed onto the selective RAPID'E. coli 2 agar (Bio-Rad, USA) supplemented with 4 μg/ml colistin (Sigma-Aldrich, USA) (4). The plates were then incubated at 37°C under aerobic conditions for 18 to 24 h. Isolates that had the expected E. coli phenotype (violet to pink colonies) were purified, and their identities were confirmed using PCR analysis that targeted an E. coli-specific 16S rRNA gene fragment (5). The E. coli exhibited resistance to important antibiotics, including colistin (polymyxin E) (Table 1), and carried the colistin resistance gene (*mcr-1.1*) on transmissible plasmids (4). To further characterize these strains, their sequence types, and other antimicrobial resistance (AMR) determinants, the genomes of two *mcr-1.1*-positive multidrug-resistant E. coli strains that exhibited high resistance to colistin were sequenced.

**TABLE 1:** Characteristics and accession numbers of genomes of the E. coli strains harboring *mcr-1.1* and isolated from water sources in Syrian refugee camps^*a*^

Isolate	Bacterial species	Source	SRA no. of raw sequences	GenBank accession no. of assembled genomes	Genome size (bp)	No. of contigs	GC content (%)	*N*_50_ (bp)	Sequencing coverage (×)	Total no. of reads	Resistance to selected antibiotics^*b*^	Colistin MIC (μg/ml)	AMR genes detected by WGS (total no. of genes)^*c*^	ST
D1	E. coli	Drinking water	SRX7741066	JACYYT000000000	5,169,139	254	50.4	74,127	82.2	2,241,796	R: PEN, AMP, AMC, LEX, GEN, KAN, STR, TET, CIP, NOR, SXT, CHL	64	*aac(3)-IId*, *aadA2*, *ant(3″)-Ia*, *aph(3″)-Ib*, *aph(3′)-Ia*, *aph(6)-Id*, *bla*_TEM-1B_, *cmlA1*, *dfrA12*, *dfrA17*, *dfrA5*, *floR*, *fosA3*, *lnu(F)*, *mcr-1.1*, *mdfA*, *mphA*, *sul1*, *sul3*, *tetA* (20 genes)	ST2936
											IR: FEP, CTX			
											S: CFM, DOR, IPM, MEM			
D3	E. coli	Well water	SRX7741068	JACYYS000000000	4,760,848	251	50.7	59,155	79.5	1,910,610	R: PEN, AMP, AMC, CTX, LEX, CFM, DOR, GEN, KAN, STR, TET, CIP, NOR, SXT, CHL	32	*aac(3)-IIa*, *aadA2*, *ant(3″)-Ia*, *aph(3′)-Ia*, *bla*_CFE-1_, *bla*_CMY-2_, *bla*_TEM-1B_, *cmlA1*, *dfrA14*, *floR*, *mcr-1.1*, *mdfA*, *mphA*, *sul3*, *tetA* (15 genes)	ST1638
											IR: FEP			
											S: IPM, MEM			

a The colistin MIC was quantified using the broth microdilution method. Resistance to other antibiotics was determined using the disk diffusion assay and the Clinical and Laboratory Standards Institute (CLSI) guidelines (16).

b R, resistance; IR, intermediate resistance; S, susceptibility; PEN, penicillin; AMP, ampicillin; AMC, amoxicillin plus clavulanic acid; FEP, cefepime; CTX, cefotaxime; LEX, cephalexin; CFM, cefixime; DOR, doripenem; IPM, imipenem; MEM, meropenem; GEN, gentamicin; KAN, kanamycin; STR, streptomycin; TET, tetracycline; CIP, ciprofloxacin; NOR, norfloxacin; SXT, trimethoprim-sulfamethoxazole; CHL, chloramphenicol. The antibiotics in the resistance profiles are arranged according to the order of antibiotics/classes listed in the CLSI guidelines (16).

c WGS, whole-genome sequencing.

For genomic DNA isolation, the E. coli strains were cultured on RAPID'E. coli 2 agar as described above. Colonies were removed from the plates with inoculation loops and suspended in the buffer supplied in the QiaAmp DNA minikit (Qiagen, USA), which was used to extract the DNA as described in the manufacturer’s protocol. DNA concentrations were determined using a Qubit BR double-stranded DNA (dsDNA) assay kit (Invitrogen, USA), and libraries were prepared with the Nextera XT DNA library preparation kit (6). The Qubit dsDNA high-sensitivity (HS) assay kit (Invitrogen, USA) was used to determine the concentration of the sample libraries, which were then diluted and denatured according to the Illumina “Denature and Dilute Libraries Guide” protocol A. The libraries were loaded into the MiSeq reagent cartridge (MiSeq reagent kit v2, 300 cycles) (6) and sequenced on a MiSeq sequencer (Illumina, USA) with the paired-end sequencing strategy (2 × 150 bp). Trimmomatic v0.36 (7) was used to remove low-quality reads. The leading three and the trailing three nucleotides were removed from the reads, and a 4-nucleotide sliding window was used to remove nucleotides from the 3′ ends when the average Phred score dropped below 20. After discarding reads shorter than 75 bp, the total number of reads was obtained for each isolate. Draft genome sequences were assembled from trimmed and filtered reads using SPAdes v3.9.0 with the “–careful” option (8). Contigs shorter than 200 bp were discarded. Evaluation of the draft genome quality was performed using QUAST v4.5 (9). Sequence types (STs) were determined using the assembled genomes and the PubMLST database (https://pubmlst.org/) with MLST software v2.16.2 (https://github.com/tseemann/mlst) (10). Default parameters were used for all software unless otherwise specified.

The properties of the draft genome sequences and their accession numbers are listed in Table 1. Using the ResFinder v3.0 database (11), it was shown that the E. coli strains carried 15 and 20 AMR genes (Table 1), including *mcr-1.1* and others that encoded resistance to important classes of antibiotics, such as aminoglycosides, diaminopyrimidines, macrolides, β-lactams, phenicols, fosfomycin, tetracyclines, fluoroquinolones, and sulfonamides. MLST analysis revealed that the strains belonged to ST2936 and ST1638. These STs have been associated with extended-spectrum β-lactamase-producing E. coli strains and those resistant to extended-spectrum cephalosporins in poultry, as well as *mcr-1*-positive E. coli, perhaps suggesting the contamination of water from nearby poultry farms (12–15).

The draft genome sequences are crucial to understanding the dissemination of AMR determinants in refugee camps, highlighting the vulnerability of these populations to resistant infections and unsanitary conditions.

### Data availability.

The assembled genome sequences were deposited under accession numbers JACYYT000000000 and JACYYS000000000.
